# Characterization of the sex determining region and development of a molecular sex identification method in a Salangid fish

**DOI:** 10.1186/s12864-024-11047-x

**Published:** 2024-11-20

**Authors:** Hao Yang, Yu-Long Li, Teng-Fei Xing, Jin-Xian Liu

**Affiliations:** 1 CAS Key Laboratory of Marine Ecology and Environmental Sciences, Institute of Oceanology, Chinese Academy of Sciences, Qingdao, 266071 China; 2 Laboratory for Marine Ecology and Environmental Science, Qingdao Marine Science and Technology Center, Qingdao, 266237 China; 3 University of Chinese Academy of Sciences, Beijing, 100049 China

**Keywords:** Salangid fish, Sex-specific loci/region, Sex determination mechanism, Genetic sex identification

## Abstract

**Background:**

The short-snout icefish, *Neosalanx brevirostris*, a member of the Salangidae family, is an economically important fishery species in China. Understanding the mechanisms underlying sex determination in this species has crucial implications for conservation, ecology and evolution. Meanwhile, there is a shortage of rapid and cost-effective genetic methods for sex identification, which poses challenges in identifying the sex of immature individuals in sex determination mechanism studies and aquaculture breeding applications.

**Results:**

Based on whole genome resequencing data, sex-specific loci and regions were found to be concentrated in a region on chromosome 2. All sex-specific loci exhibited excess heterozygosity in females and complete homozygosity in males. This sex determining region contains seven genes, including cytochrome P450 aromatase *CYP19B*, which is involved in steroidogenesis and is associated with 24 sex-specific loci and two W-deletions. A haploid female-specific sequence was identified as paralogous to a diploid sequence with a significant length difference, making it suitable for rapid and cost-effective genetic sex identification by traditional PCR and agarose gel electrophoresis, which were further validated in 24 females and 24 males with known phenotypic sexes.

**Conclusions:**

Our results confirm that *N.* *brevirostris* exhibits a female heterogametic sex determination system (ZZ/ZW), with chromosome 2 identified as the putative sex chromosome containing a relatively small sex determining region (~ 48 Kb). The gene *CYP19B* is proposed as a candidate sex determining gene. Moreover, the development of PCR based method enables genetic sex identification at any developmental stage, thereby facilitating further studies on sex determination mechanisms and advancing aquaculture breeding applications for this species.

**Supplementary Information:**

The online version contains supplementary material available at 10.1186/s12864-024-11047-x.

## Background

Sex determination (SD) has long stood as a classic and challenging topic in life sciences, which serves as a master switch to bipotential gonadal primordium and activates the sex differentiation pathway [1]. Unlike the highly conserved SD systems observed in mammals and birds, fishes exhibit a remarkable diversity of SD systems, sex chromosomes and SD genes, making them an ideal model for studying the evolution of SD mechanisms in vertebrates [2]. Fishes cover nearly all known types of SD in vertebrates, including genetic sex determination (GSD), environmental sex determination (ESD) or a combination of both [3]. Most cases of GSD of teleosts fall into two main categories: female heterogametic (ZZ/ZW system, e.g., *Protosalanx hyalocranius* [4], *Trachinotus anak* [5]) and male heterogametic (XX/XY system, e.g., *Clupea harengus* [6], *Solea senegalensis* [7]). Additionally, within GSD systems, master sex determining (MSD) genes in teleosts exhibit considerable variation, including classical transcription factors such as *DMY* [8, 9], *SOX2* [10] or *SOX3* [11]; transforming growth factor β (TGF-β) signaling genes such as *GSDF* [12] or *AMH* [13, 14] and its receptor *AMHR2* [15]; genes related to the steroidogenic pathway such as *BCAR1* [16], *HSD17B1* [17] or *CYP19A* [18]; and some unexpected genes like the interferon-related *SDY* gene [19]. The origins of these MSD genes typically involve either gene duplication followed by sub- or neo-functionalization or allelic diversification [20]. However, there appears to be no universal MSD gene among fishes, even among closely related species [21], and SD genes in most fishes are still unclear.

The identification of sex-specific loci and regions is essential for revealing SD systems, understanding SD mechanisms, and ultimately pinpointing SD genes [4, 22]. Furthermore, in most fishes, sexual characteristics often become evident only in adulthood or upon reaching sexual maturity, making early-life sex identification difficult, especially in economically important species. Compared to traditional methods such as cytogenetic and histological analysis, developing sex-specific molecular markers provides a rapid and cost-effective approach of sex identification [23]. Traditional techniques for screening sex-specific markers, including amplified fragment length polymorphism (AFLP) [24], simple sequence repeat (SSR) [25], and randomly amplified polymorphic DNA (RAPD) [26], are often inefficient and expensive. Recently, next generation sequencing (NGS) has revolutionized SD research by enabling cost-effective identification of sex-specific markers on a whole genome scale. This approach has been successfully applied in some fish species such as *Collichthys lucidus* [27], *Protosalanx hyalocranius* [28], and *Spinibarbus hollandi* [23], etc.

The short-snout icefish, *Neosalanx brevirostris*, belongs to the Salangidae family and mainly inhabits coastal and estuarial regions of China, as well as rivers and lakes of the Yangtze River and Huai River [29, 30]. The *N. brevirostris* is an important commercial fishery species, especially in inland lakes, and has been extensively introduced into lakes and reservoirs in southern China for aquaculture purposes [31]. Established artificial breeding techniques have enhanced aquaculture yields by facilitating the release of zygotes. As other Salangid fishes, *N. brevirostris* follows an annual life cycle and dies after spawning. Sexual dimorphism in Salangid fishes emerges at maturity, characterized by: (i) a single row of scales at the base of the anal fin of males, which absent in females; (ii) greater anal fin height in males; (iii) longer and pointed first ray of the pectoral fin in males; (iv) greater body height at the anus in males [32]. However, discernible phenotypic differences between sexes are absent before sexual maturation. At present, a rapid and cost-effective genetic sex identification method is still lacking, posing challenges in identifying the sex of immature individuals in ecological and evolutionary studies, and aquaculture breeding programs. Meanwhile, apart from *Protosalanx hyalocranius* [4], there have been no studies on SD systems and genes in Salangid fishes. A previous cytogenetic study of *N. brevirostris* indicated the absence of heteromorphic chromosomes [33]. Recently, we constructed a chromosome-level genome assembly for *N. brevirostris* (accession link: https://figshare.com/s/c056fdfb62dbeab041f9), spanning 442 Mb with high continuity (contig N50 = 5.3 Mb) and integrity (BUSCO score = 96% based on actinopterygii odb10 dataset), which provided a valuable genetic resource to elucidate SD mechanisms and sex chromosomes evolution in Salangid fishes.

To study the SD genetic mechanism in *N. brevirostris*, whole genome resequencing and genome-wide association analyses were conducted on 44 mature individuals (20 males and 24 females). Sex-associated and sex-specific loci/regions were isolated to address three key objectives: (i) elucidating the SD system of this species, (ii) characterizing the putative sex determining region (SDR) and pinpointing candidate SD genes, and (iii) developing a rapid and cost-effective molecular method for genetic sex identification. These findings will hold promise for enhancing our understanding of SD mechanisms and provide insights into the evolutionary aspects of early differentiation of primitive sex chromosomes in teleosts.

## Materials and methods

### Sampling, sex identification and sequencing

During the spawning season, a total of 44 sexually mature individuals of *N. brevirostris* were collected from the landlocked wild population in Hongze Lake (8 males and 12 females) and Taihu Lake (12 males and 12 females) in 2022. The sex of each fish was determined by visual inspection of the secondary sexual characteristics (Supplementary Fig. S1). Muscle tissues were kept individually in 95% ethanol and stored at −80℃. Genomic DNA was extracted following the standard phenol–chloroform extraction method. DNA extracts were visualized on 1% agarose gels to assess quality and were subsequently quantified using Qubit fluorometer. Whole genome resequencing libraries with insert size ~ 350 bp were constructed and then sequenced on the DNBSEQ-T7 platform using 150-bp paired-end sequencing with a minimum coverage of ~ 20X for each individual. In addition to the 44 individuals used for whole genome resequencing, genomic DNA from another 48 samples collected in 2023 with known phenotypic sex (12 males and 12 females each from Hongze Lake and Taihu Lake) was extracted to test the effectiveness and efficiency of the PCR based sex identification method.

### Data filtering and genotyping

For raw reads, adaptors and low-quality reads were removed using FASTP v0.23.2 [34] with default parameters. The generated clean reads were mapped to the chromosome-level genome of *N. brevirostris* using BWA-MEM v0.7.17 [35] with default parameters. Aligned BAM files were sorted using Sambamba v1.0.1 [36], and PCR duplicates were marked using Samblaster v0.1.26 [37]. Single nucleotide polymorphism (SNP) calling was performed by BCFtools v1.10.2 in SAMtools v1.10 [38] based on a Bayesian framework, and SNPs were stored in a VCF file. To retain high-quality SNPs for the downstream analysis, we used the following criteria to filter all SNPs identified: (a) only biallelic SNPs were retained; (b) SNP overall quality score (Q) ≥ 30 and genotype quality (GQ) ≥ 20; (c) minimum coverage depth for each individual of each SNP site ≥ 7; (d) SNP were called in at least 90% individuals overall and 12 individuals for each group (sex); and (e) global minor allele frequency (MAF) ≥ 0.05, and (f) retain the SNP with local MAF ≥ 0.2 in any group (sex) but failed to meet the criteria of the global MAF ≥ 0.05. SNPs VCF file was converted into other formats using PLINK v1.90b6.21 [39] or in-house Perl script. The density plot of SNPs across whole genome was plotted using R package CMplot v4.5.1 [40].

### Sex-associated SNPs detection

To detect SNPs significantly associated with sex, we employed two distinct methods for genome-wide association testing. Initially, Fisher's exact test (FET) was conducted based on PLINK, where sexes were encoded as case/control phenotypes. Secondly, we utilized the p*F*_ST_ function from the vcflib software library (accession link: https://github.com/vcflib) to assess allele frequency differentiation between populations defined by sex. Subsequently, *p*-values from both methods were corrected using false discovery rate (FDR) correction, with an FDR threshold of 0.05 for significance determination.

### Sex-specific SNPs discovery

To identify the putative SD system, we utilized an in-house Perl script (accession link: https://github.com/lyl8086/find_sex_loci) to isolate SNPs that exclusively exhibited a heterozygous genotype in one sex group. For an XX/XY SD system, genomic regions were identified where SNPs were heterozygous in males while corresponding female SNPs were homozygous. Conversely, for a ZZ/ZW SD system, heterozygous SNPs were expected in females, with corresponding male SNPs being homozygous. Due to potential missing data and low coverage depth, some heterozygous SNPs might be miscalled as homozygous, necessitating a minimum of 18 heterozygotes in one sex group (with overall observed heterozygosity ≥ 0.75) and no heterozygotes in the other sex group (i.e., all SNPs were homozygous for one allele). These SNPs were extracted using genotypes information processed by VCFtools v0.1.15 [41] and were defined as putative sex-specific SNPs. Linkage disequilibrium (LD) measurements (*r*^2^) for these SNPs were also computed using VCFtools. Notably, the presence of heterozygosity in one sex and its absence in the other is insufficient alone to definitively support either an XY or ZW system. While all male heterozygosity could suggest an XY system, it would also indicate a ZW system with a W-deleted region, and the SNPs were segregating variations. Therefore, we additionally conducted coverage analysis on these sex-specific loci by calculating the depth of each SNP in each individual.

### Female-specific genomic regions identification

The individual used for reference genome sequencing and assembly was identified as a heterogametic female (ZW), making the reference genome suitable for identifying female-specific genomic regions. Firstly, clean reads from all individuals were aligned to this reference genome using BWA-MEM. Secondly, the depth of coverage for each individual was extracted with SAMtools, and differences between sexes were evaluated using Welch’s t-test. This analysis was performed by an in-house Perl script (accession link: https://github.com/lyl8086/find_sex_loci). Thirdly, genome loci showing significant depth of coverage differences (*p*-value < 1e-4) between sexes were identified. For female-specific genomic regions, we required that the sequencing depth of the female individuals should be approximately half of the average sequencing depth across the genome, while the sequencing depth for male individuals should be extremely low (≤ 2X).

### Structural variant calling

We utilized a long-read-based approach for structural variant (SV) calling to produce high-confidence SV calls. Firstly, the CCS reads from the female reference individual were aligned to the reference genome using pbmm2 v1.13.1 [42] to generate the alignment BAM file. The pbsv v2.9.0 (accession link: https://github.com/PacificBiosciences/pbsv) discover module was then employed to identify structural variation signatures within the aligned BAM, followed by the call module to call SVs. Finally, alignment files for the sex-specific genomic regions were meticulously examined using Integrative Genomics Viewer (IGV) v2.16.1 [43].

### Gene annotation

All sex-specific loci were annotated with snpEFF v5.2c [44] according to the genome annotation file. The snpEFF assigned properties such as gene name and consequence (e.g., missense, synonymous, etc.) to each SNP. Upstream and downstream variants were defined as SNPs located within 5 kb in physical distance from a gene. Gene predictions located in the female-specific genomic regions were also extracted from the annotation file.

### Development and validation of a sex-specific marker

We extracted all the female-specific genomic region sequences and employed local BLASTn from BLAST_+_ v2.11.0 [45] for homologous alignment across the whole genome. A 590 bp female-specific genomic region on W chromosome was found paralogous to another genomic region shared by both females and males with a length difference, suggesting an ideal system for rapid genetic sex identification by traditional PCR and gel electrophoresis. A primer pair (forward: 5’-TGCTCTTGCCAAAACACTGC-3’; reverse: 5’-GGGATTTGGTGTCTGGCAGA-3’) was designed based on the consensus flanking sequence of the female-specific genomic region and its paralogous genomic region using Primer Premier 5.0 software (accession link: http://www.premierbiosoft.com/). The DNA samples of eight females and eight males were used for the validation of effectiveness of the primers in discriminating sexes. Each PCR reaction was carried out in a final volume of 25 µl containing 1 µl DNA template, 12.5 µl 2xTaqMasterMix (Dongsheng Biotech Co., China), 0.5 µl each primer (10 µM) and 10.5 µl ddH_2_O. PCR cycling conditions were as follows: pre-denaturation at 95 °C for 4 min; 35 cycles of denaturation at 95 °C for 1 min, annealing at 58 °C for 1 min, and extension at 72 °C for 1 min; a final extension of 10 min at 72 °C. PCR products were loaded onto a 2% agarose gel and subjected to electrophoresis at 70 V for 45 min. To validate the accuracy of the sex identification method, a second round of verification was conducted using 48 mature individuals with known phenotypic sexes. For further confirmation of the amplified sequences, PCR products from one female and one male were sent to Sangon Biotech Co., Ltd. for Sanger sequencing.

## Results

### Whole genome resequencing

We performed whole genome resequencing of 44 sexually mature short-snout icefish individuals from Hongze Lake (8 males and 12 females) and Taihu Lake (12 males and 12 females), with an average depth of 29X (ranging from 20 to 41X) (Supplementary Table S1). After aligning the resequencing data to the reference genome and subsequent filtering, we identified a total of 2,313,554 high-quality bi-allelic SNPs distributed across 28 chromosomes (Supplementary Fig. S2). Analysis of these SNPs revealed an overall Weir and Cockerham weighted *F*_ST_ of −0.00054, suggesting no significant genetic differentiation between the two sex groups at the whole genome level.

### Identification of putative sex chromosome and characterization of sex determining region

**Fig. 1:**
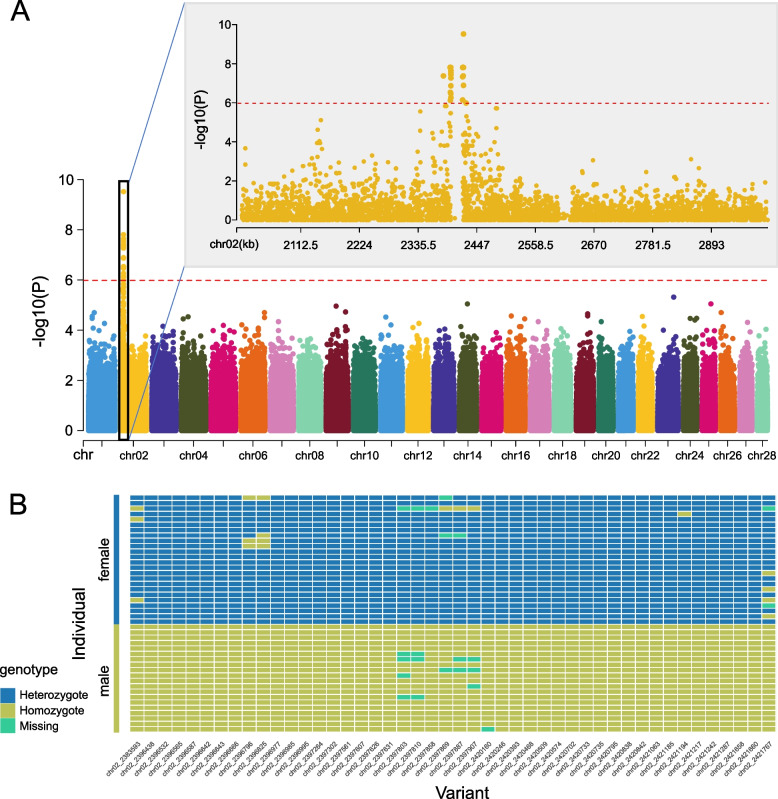
Characterization of sex-associated SNPs and sex-specific SNPs. **A** Manhattan plot for *p*-value. The x-axis represents the 28 chromosomes, whereas the y-axis represents *p*-value from Fisher’s exact test in -log_10_ scale. The red dashed-line represents the genome-wide significant associated line, where *p* = 1.03e-6. The black solid-line box highlights the region that is significantly associated with sex with a zoomed-in view on the side. **B** Heatmap shows the homozygous or heterozygous status of each individual in each sex group at sex-specific SNPs

**Fig. 2:**
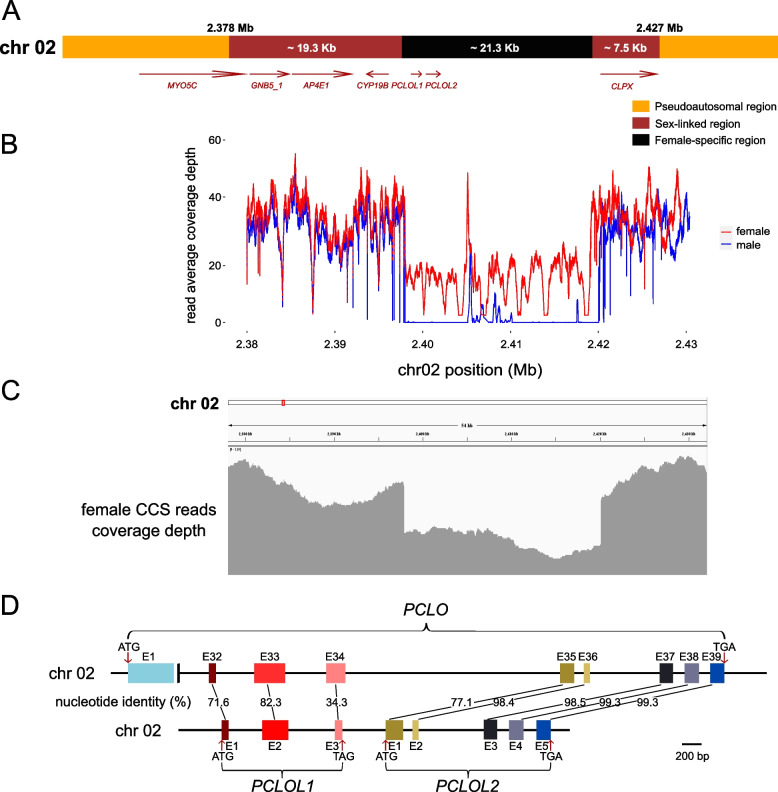
Sex determining region in *Neosalanx brevirostris*. **A** Distribution of female-specific region (black), sex-linked region (brown), and flanking pseudoautosomal region (orange). Gene annotation results are also displayed. The arrow represents the direction of the gene. **B** Average coverage depth of male (blue line) and female (red line) resequencing reads on chr02 (2.38—2.43 Mb). **C** Alignment of the CCS reads from a female individual to the ZW genome revealed a W-specific region in the sex determining region. **D** Gene structure and sequence identity between *PCLO* and *PCLOL1*/*PCLOL2*. Exons are represented by boxes with shared percentage nucleotide identity indicated and introns are represented by black lines

To identify the putative sex chromosome and characterize the SDR of *N. brevirostris*, we examined sex-biased signals, including sex-linked SNPs and coverage differences between sexes. After applying FDR correction, 50 SNPs were detected by FET and 61 SNPs by p*F*_ST_ as significantly associated with sex. Of these, 50 SNPs were overlapped between the two tests (Fig. 1A). A total of 46 SNPs were identified as sex-specific SNPs, showing excess heterozygosity in females (*H*_O_ ranged from 0.818 to 1.000, with a mean of 0.983) and all homozygosity in males (Fig. 1B and Supplementary Table S2). To minimize false positives, we selected these 46 common SNPs for subsequent analysis (Supplementary Fig. S3). All these SNPs were located exclusively within two contiguous segments on chromosome 2, which we defined as sex-linked regions, including their 5 kb upstream and downstream flanking areas (~ 19.3 kb and ~ 7.5 kb, respectively) (Fig. 2A). These SNPs displayed high *F*_ST_ values between sexes (ranging from 0.392 to 0.576) (Supplementary Table S2) and strong LD (mean *r*^2^ = 0.946). No significant difference in depth of coverage between sexes was observed for the 46 sex-specific SNPs, with mean depths of 40.781 (sd = 4.607) in females and 34.657 (sd = 4.675) in males (Supplementary Table S3). Interestingly, a single female-specific region (~ 21.3 Kb, positions 2,397,919—2,419,244 bp) was found between the two sex-linked regions (Fig. 2A) based on coverage analysis of male and female resequencing reads (Fig. 2B), which contained high content of repetitive sequences (~ 56%). The results of structural variation and visualization of CCS reads alignment further confirmed this discovery (Fig. 2C). These two features, where females carry additional genomic sequences, with co-located sequence divergence seen in the form of heterozygous variants, are indicative of a ZZ/ZW SD system. Taken together, our findings confirmed that chromosome 2 functions as a putative sex chromosome in *N. brevirostris*, with a relatively small SDR (~ 48 Kb), including two sex-linked regions and one female-specific region in between (Fig. 2A).

### Genes in the sex determination region

We annotated seven genes in the SDR according to the genome annotation file (Fig. 2A). Of the 46 sex-specific SNPs, 22 SNPs were located in genic regions, including one in exon, 16 in introns, and five in UTRs (Supplementary Table S4). In one sex-linked region, the sole SNP located in an exon was found in the *CYP19B* gene as a synonymous mutation. Besides, structure variant analysis revealed four small insertions in the SDR beyond the previously mentioned female-specific region (Supplementary Table S5). Three of them appear almost exclusively on the Z chromosome, while the other is primarily on the W chromosome, suggesting a high probability of three W-deletions and one W-insertion. Two of the three W-deletions are associated with the *CYP19B* gene, one (pbsv_5700) is located in an intron and the other (pbsv_5701) is 23bp upstream of the start codon. Three other genes *MYO5C*, *GNB5_1* and *AP4E1* show positional conservation with *CYP19B* across various fish species [46]. In the other sex-linked region, only one gene *CLPX* was annotated.

Furthermore, two Piccolo-like genes were identified (referred as *PCLOL1* and *PCLOL2*) in the ~ 21.3 Kb female-specific region (positions 2,398,942—2,400,174 bp, Gene ID: Nbr_001519-T1; positions 2,400,617—2,402,303 bp, Gene ID: Nbr_001520-T1). Another Piccolo gene *PCLO*, also located on chromosome 2 (positions 5,385,203—5,439,211 bp, Gene ID: Nbr_001691-T1) was found as diploid in both males and females. However, *PCLOL1* and *PCLOL2* were annotated de novo and lacked supporting evidence from RNA-seq data. The canonical *PCLO* gene contains 39 exons, whereas *PCLOL1* and *PCLOL2* have only three and five exons, respectively, suggesting that the latter may be undergoing a process of genetic degeneration that results in the loss of most exons and the absence of complete gene structure (Fig. 2D). The eight exons of *PCLOL1* and *PCLOL2* align with the last eight exons of the canonical *PCLO* gene, exhibiting nucleotide identities ranging from 34.3% to 99.3% (average = 85.8%) (Fig. 2D).

### Design and verification of primers for genetic sex identification

**Fig. 3:**
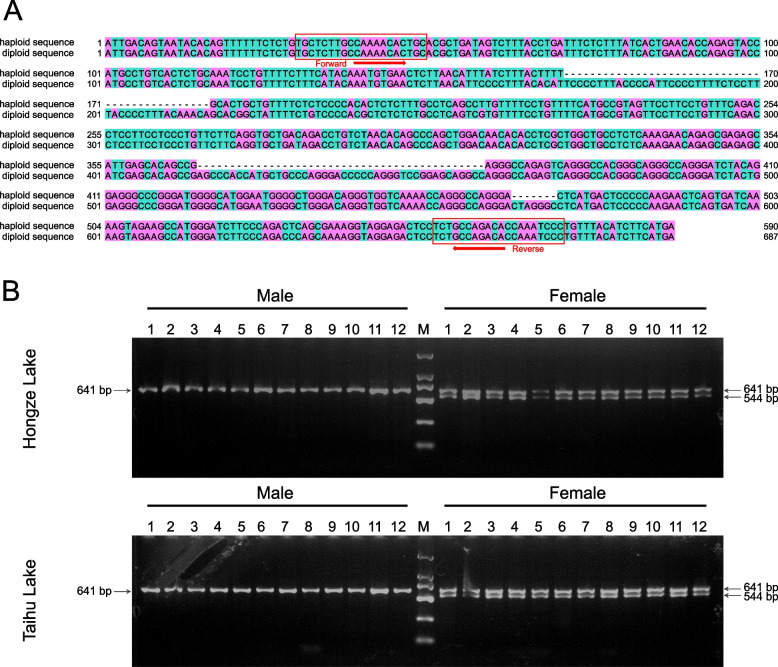
Sex-specific marker design and verification. **A** Alignment of the haploid sequence (female specific: chromosome 2 from 2,399,081 to 2,399,670 bp) and diploid sequence (female and male shared: chromosome 2 from 5,433,952 to 5,434,638 bp). The primers are displayed in the red frame. **B** PCR amplification results of 48 individuals (12 males and 12 females each from Hongze Lake and Taihu Lake). The 641 bp longer band (shared by males and females) and the shorter 544 bp female specific band are indicated, respectively. The DL 2000 DNA marker is shown in the middle. The original gel electrophoresis images are presented in Supplementary file Fig. S4 and Fig. S5

DNA sequence alignment between the haploid female-specific genomic region (chromosome 2: 2,399,081—2,399,670 bp) and its paralogous diploid counterpart (chromosome 2: 5,433,952—5,434,638 bp), unveiled three gaps, amounting to 97 bp (Fig. 3A), making them ideal system for rapid and cost-effective genetic sex identification. Primers designed in the highly conserved flanking regions of these sequences amplified two target sequences of different lengths (641 and 544 bp) in females and a single 641 bp amplicon in males. PCR of eight female and eight male individuals confirmed the presence of two bands in females and one in males. Sanger sequencing of PCR products from one female and one male confirmed the sequences. Subsequent validation using 48 individuals with known phenotypic sex (12 males and 12 females each from Hongze Lake and Taihu Lake) consistently showed two bands in females and one in males (Fig. 3B). The PCR yield of the shorter band unique to females was less than that of the longer band, confirming the haploid nature of the female-specific sequence and the diploid nature of its paralogous sequence in both sexes. Thus, the primers represent an ideal tool for rapid and cost-effective genetic sex identification in *N. brevirostris*.

## Discussion

More than 30,000 fish species inhabit a wide range of aquatic habitats worldwide [47], yet SD genes have been identified in only 114 species so far [20], leaving the SD mechanism in most fish a mystery. Our research highlights the transformative power of advanced sequencing technologies and population genomics approaches in uncovering and characterizing sex chromosome and sex-linked regions in non-model species. In this study, we employed whole genome resequencing and sex-biased signals analyses to identify the SD system, the putative sex chromosome and candidate SD gene in *N. brevirostris*. This work aims to elucidate the SD mechanism specific to this species and provide valuable genetic resources for further investigations on sex chromosome evolution in Salangid fishes.

Cytologically differentiated sex chromosomes are commonly observed in mammals and most birds [48, 49]. However, the absence of cytologically differentiated sex chromosomes in most teleosts does not preclude the presence of a differentiated SDR at the molecular level. For instance, relatively large SDRs have been documented in species with morphologically undifferentiated sex chromosomes, such as *Sarotherodon melanotheron* (~ 17.9 Mb) [50], *Carassius auratus* (~ 11.7 Mb) [51], and *Oreochromis niloticus* (~ 10.7 Mb) [52]. Conversely, small SDRs also have been identified through whole genome analyses in *Clupea harengus* (~ 0.3 Mb) [6], *Perca flavescens* (~ 0.1 Mb) [53], and *Phyllopteryx taeniolatus* (~ 47 Kb) [54]. In *Takifugu rubripes*, the SDR is minimal, consisting only of a missense SNP on the Y chromosome [55]. In *N. brevirostris*, we detected a relatively small SDR (~ 48 Kb) on chromosome 2, and a ZZ/ZW SD system was indicated. This region is notably enriched with sex-specific SNPs and repeated sequences, as well as a large W-specific insertion, potentially playing a role in recombination suppression between Z and W.

As many currently characterized MSD genes in teleosts belong to a few function families (e.g., transcription factor, TGF-β signaling, and steroidogenesis), sex-specific allelic variants or duplicate gene members within these families are compelling candidates for potential MSD genes [20]. Our results indicated that *CYP19B*, a gene of interest within the cytochrome P450 family, is putatively involved in SD of *N. brevirostris*. Cytochrome P450 aromatase (*CYP19*) serves as the terminal enzyme in the steroidogenic pathway, converting androgens (e.g., testosterone) into estrogens (e.g., estradiol) [56]. The sex-specific differential expression of *CYP19* regulates the ratio of androgens to estrogens, making its appropriate expression crucial during the critical period of ovarian differentiation [57]. Due to the third round of whole genome duplication event specific to the teleost lineage, the *CYP19* gene underwent duplication, giving rise to the paralogs *CYP19A* and *CYP19B* [58]. Despite their similar enzymatic activities, some divergences in their gene promoter sequences have supported their differential tissue expression and regulation across various fish species [58]. There are several studies identifying *CYP19* as potential SD gene in teleosts. In *Danio rerio*, all *CYP19A* mutants and *CYP19A*/*CYP19B* double mutants were observed to be male [59]. Recently, a case of a *CYP19A* duplicate evolving into the MSD gene has been documented in *Pseudocaranx georgianus* [18]. In *Colossoma macropomum*, significant sex-specific differences in *CYP19B* expression prior to sex differentiation have been observed [46]. In the present study, the only sex-specific SNP located in an exon was found in the *CYP19B* gene, which caused a synonymous mutation. At this SNP, all females were heterozygous (A/G), while all males were homozygous (A/A), compatible with the proposed ZZ/ZW SD system. In absence of nonsynonymous substitutions of *CYP19B*, further work may investigate whether *CYP19B* expression differed between genetic males and females during early development. Additionally, a W-deletion in the intron and another W-deletion along with 23 sex-specific SNPs in the upstream region have been identified as linked to *CYP19B*, potentially functioning as transcriptional regulatory elements. In *Scophthalmus maximus*, a diagnostic variant outside the coding region of the candidate SD gene *SOX2* was identified, which is responsible for differential expression between sexes [10]. Noteworthy, several genes potentially involved in steroidogenesis have also been characterized as MSD genes, including *HSD17B1* in *Seriola quinqueradiata* [17], *SULT1ST6Y* in *Thunnus orientalis* [60], *BCAR1* in *Ictalurus punctatus* [16].

The *N. brevirostris* is widely distributed in both fresh and brackish waters across China and has been artificially introduced to numerous lakes and reservoirs due to its high commercial value [31]. However, due to a lack of morphological distinctions during most stages of its life cycle, distinguishing males and females based on secondary sexual characteristics is only feasible upon sexual maturity. Consequently, the PCR based method developed based on a haploid female-specific sequence and it paralogous diploid counterpart in our study is essential for genetic sex identification in early life stage, which is important for ecological studies and aquaculture breeding applications. Noteworthily, our primer can amplify two bands in females and one band in males, and the size difference between the two bands produced in females effectively minimizes the risk of false negatives in genotyping. Concurrently, investigating the differential expression of sex-linked or SD genes in both sexes is a prerequisite for elucidating the molecular mechanisms governing SD and development. Given that sexual dimorphism is not apparent during embryo, larval, and juvenile stages, the development of a PCR based sex identification tool is imperative for accurately discerning genetic sex. Thus, the convenient method developed in the present study will greatly facilitate further exploration of the molecular mechanisms underlying SD in *N. brevirostris*.

## Conclusions

Our results showed that *N. brevirostris* exhibits a female heterogametic (ZZ/ZW) system, with SD in this species governed by genetic factors. We characterized a relatively small SDR of approximately 48 Kb on chromosome 2, identifying chromosome 2 as the putative sex chromosome. The gene *CYP19B* associated with 24 sex-specific SNPs and two W-deletions, which is involved in steroidogenesis, is proposed as a strong candidate SD gene for *N. brevirostris*. Further studies are needed to validate the function of *CYP19B* in SD of *N. brevirostris*. Additionally, we discovered a female-specific haploid sequence that is paralogous to a diploid sequence. Leveraging these sequences, we developed a rapid and cost-effective PCR-based method for genetic sex identification, which could facilitate the elucidation of SD molecular mechanisms and advance breeding technologies for this species.

## Supplementary Information

Supplementary Material 1.

## Data Availability

The datasets that support the findings of this study are openly available in the NCBI Sequence Read Archive (SRA) under BioProject accession No. PRJNA1140889 for whole genome resequencing data and No. PRJNA1076684 for CCS HiFi data. The genome assembly and annotation files for *Neosalanx brevirostris* are publicly available at figshare, accession link: https://figshare.com/s/c056fdfb62dbeab041f9.

## Abbreviations

SD: Sex determination
GSD: Genetic sex determination
ESD: Environmental sex determination
MSD: Master sex determining
TGF-β: Transforming growth factor β
AFLP: Amplified fragment length polymorphism
SSR: Simple sequence repeat
RAPD: Randomly amplified polymorphic DNA
NGS: Next generation sequencing
SDR: Sex determining region
SNP: Single nucleotide polymorphism
MAF: Minor allele frequency
FET: Fisher’s exact test
FDR: False discovery rate
LD: Linkage disequilibrium
SV: Structural variant
IGV: Integrative genomics viewer
