# Supplementary material for: Characterization of the sex determining region and development of a molecular sex identification method in a Salangid fish
Source: BMC Genomics. 2024 Nov 20;25:1120. doi: 10.1186/s12864-024-11047-x (PMC11580623; doi:10.1186/s12864-024-11047-x)
Supplement: Supplementary file 1 — Supplementary Material 1. [file 12864_2024_11047_MOESM1_ESM.docx]

***Supplementary Materials* for:** **Characterization of the sex determining region and development of a molecular sex identification method in a Salangid fish**

Hao Yang^1,2,3^, Yu-Long Li^1,2,*^, Teng-Fei Xing^1,2^, and Jin-Xian Liu^1,2,*^

^1^ CAS Key Laboratory of Marine Ecology and Environmental Sciences, Institute of Oceanology, Chinese Academy of Sciences, Qingdao 266071, China

^2^ Laboratory for Marine Ecology and Environmental Science, Qingdao Marine Science and Technology Center, Qingdao 266237, China

^3^ University of Chinese Academy of Sciences, Beijing 100049, China

^*^ Correspondence: Yu-Long Li: [lyl@qdio.ac.cn](mailto:lyl@qdio.ac.cn;), Jin-Xian Liu: [jinxianliu@gmail.com](mailto:jinxianliu@gmail.com).

## Supplementary Figures

**
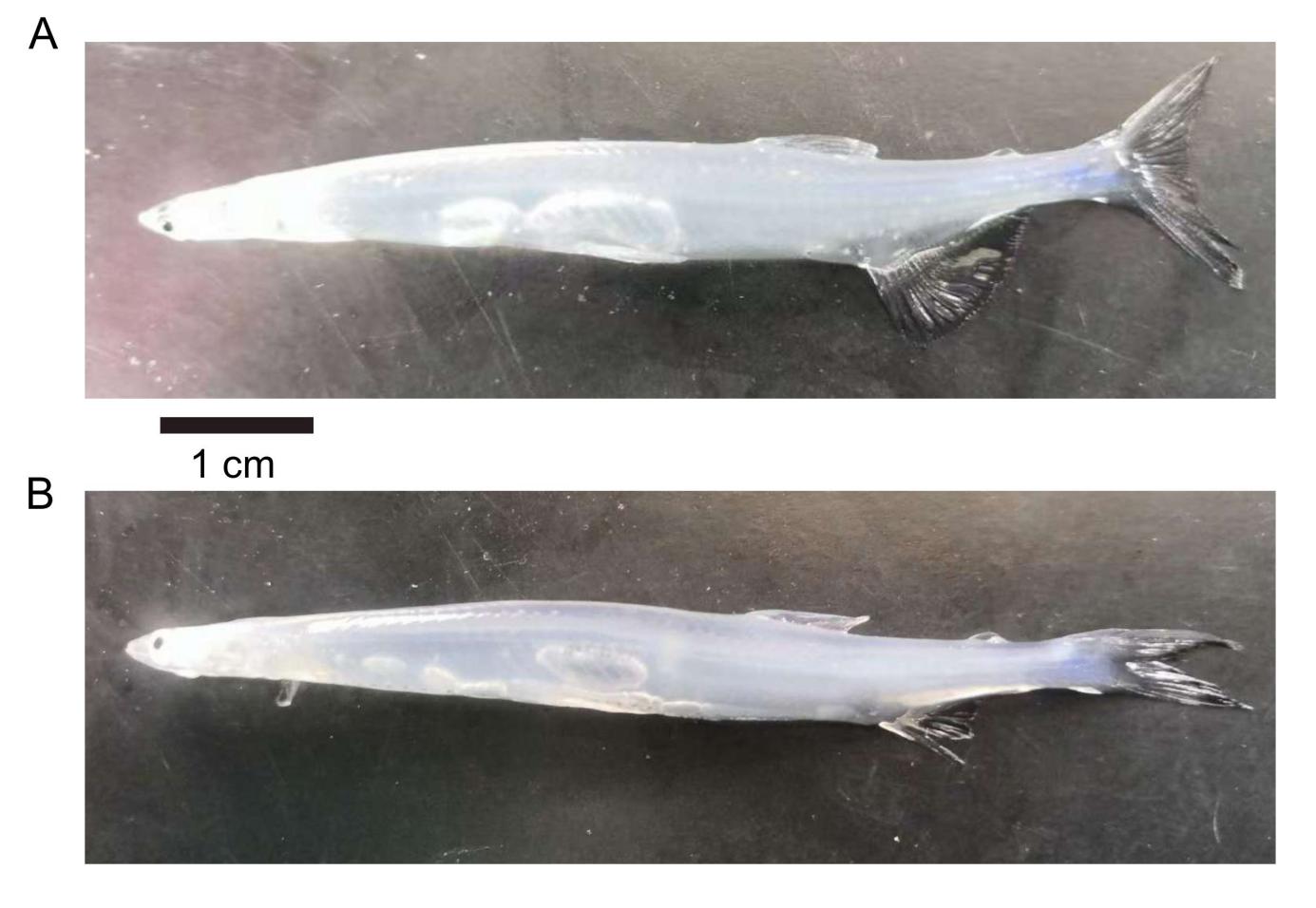
**

**Fig. S1.** Main view of male (A) and female (B) sexually mature individual of *Neosalanx brevirostris*.


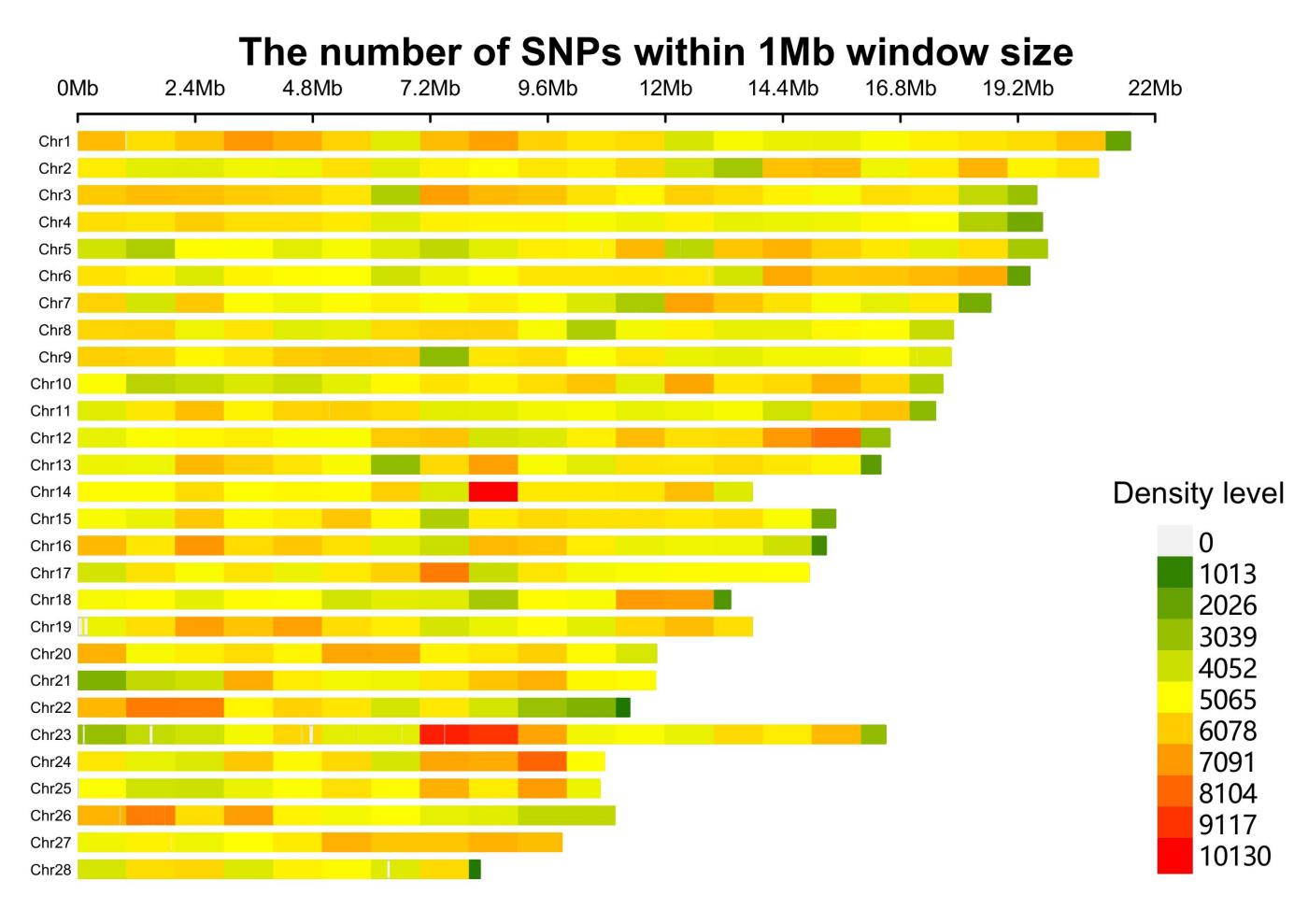


**Fig. S2.** Density plot of SNPs identified on the 28 chromosomes of the *Neosalanx brevirostris* genome.


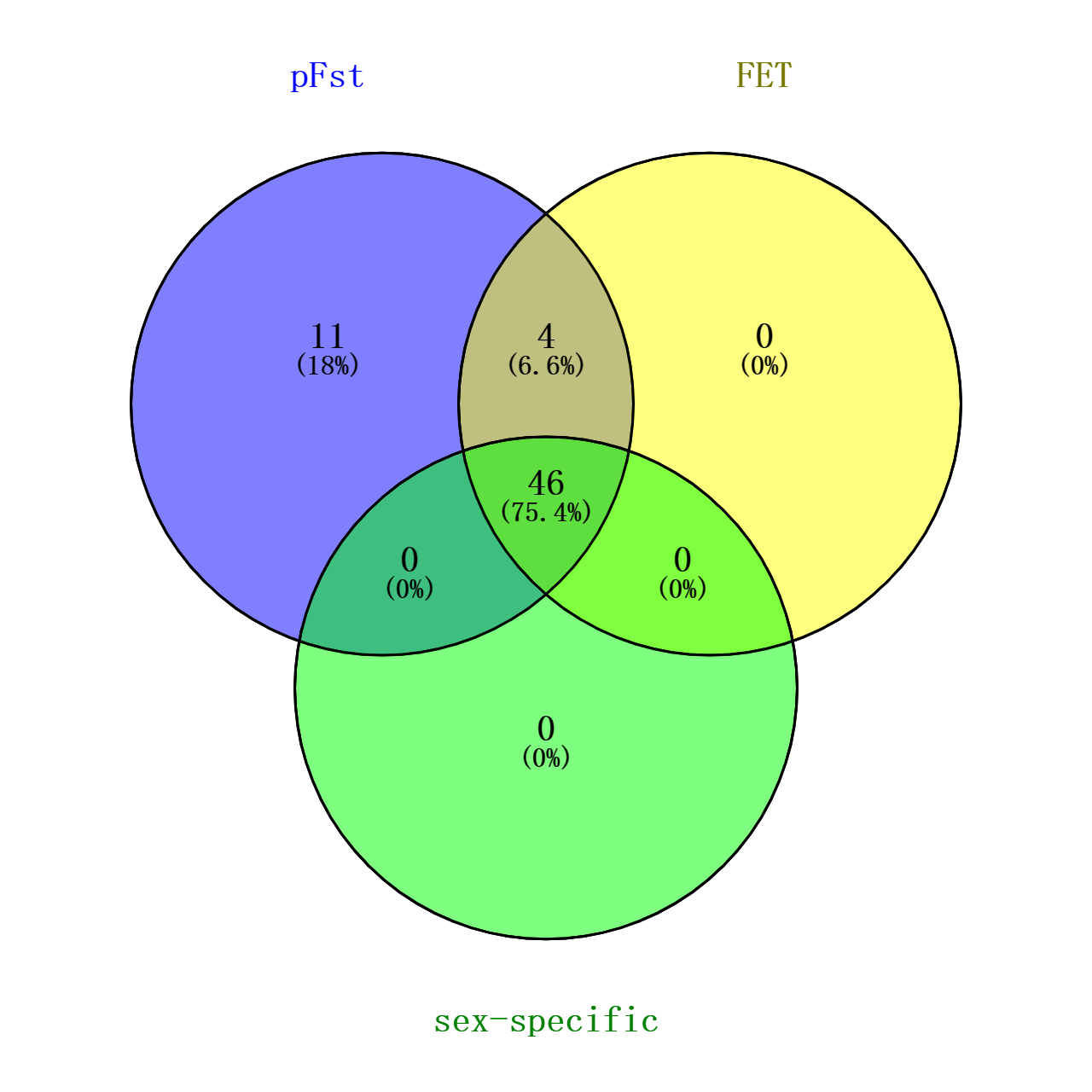


**Fig. S3.** Overlap among sex-linked SNPs detected by p*F*_ST_, Fisher’s exact test and a sex-specific testing. FET: Fisher’s exact test.

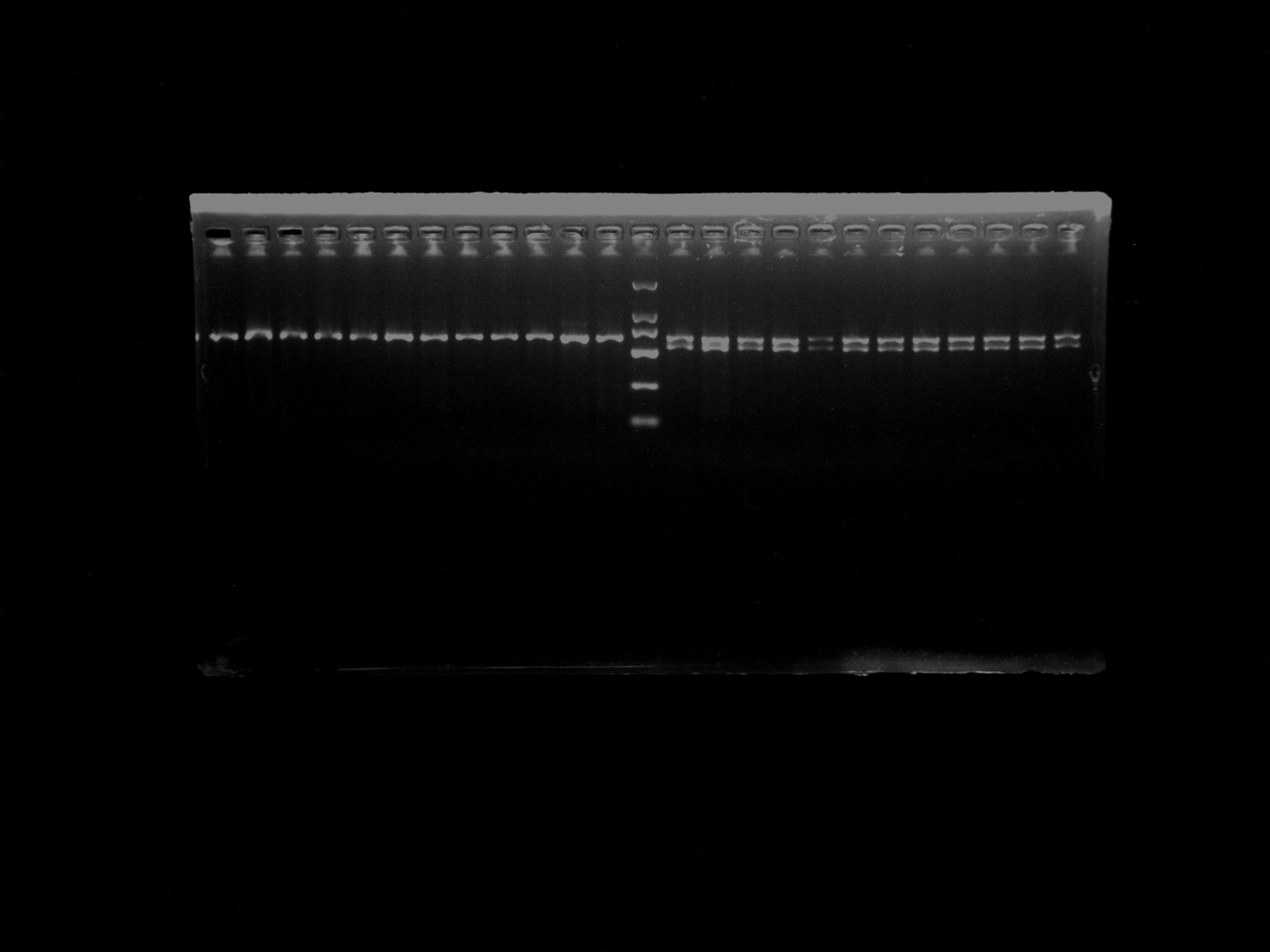


**Fig. S4.** The original gel electrophoresis image of PCR amplification results of 24 individuals from Hongze Lake. The DL 2000 DNA marker is shown in the middle. The 12 male individuals with a single band (641 bp) are shown on the left and the 12 female individuals with two bands (641 bp and 544 bp) are shown on the right.


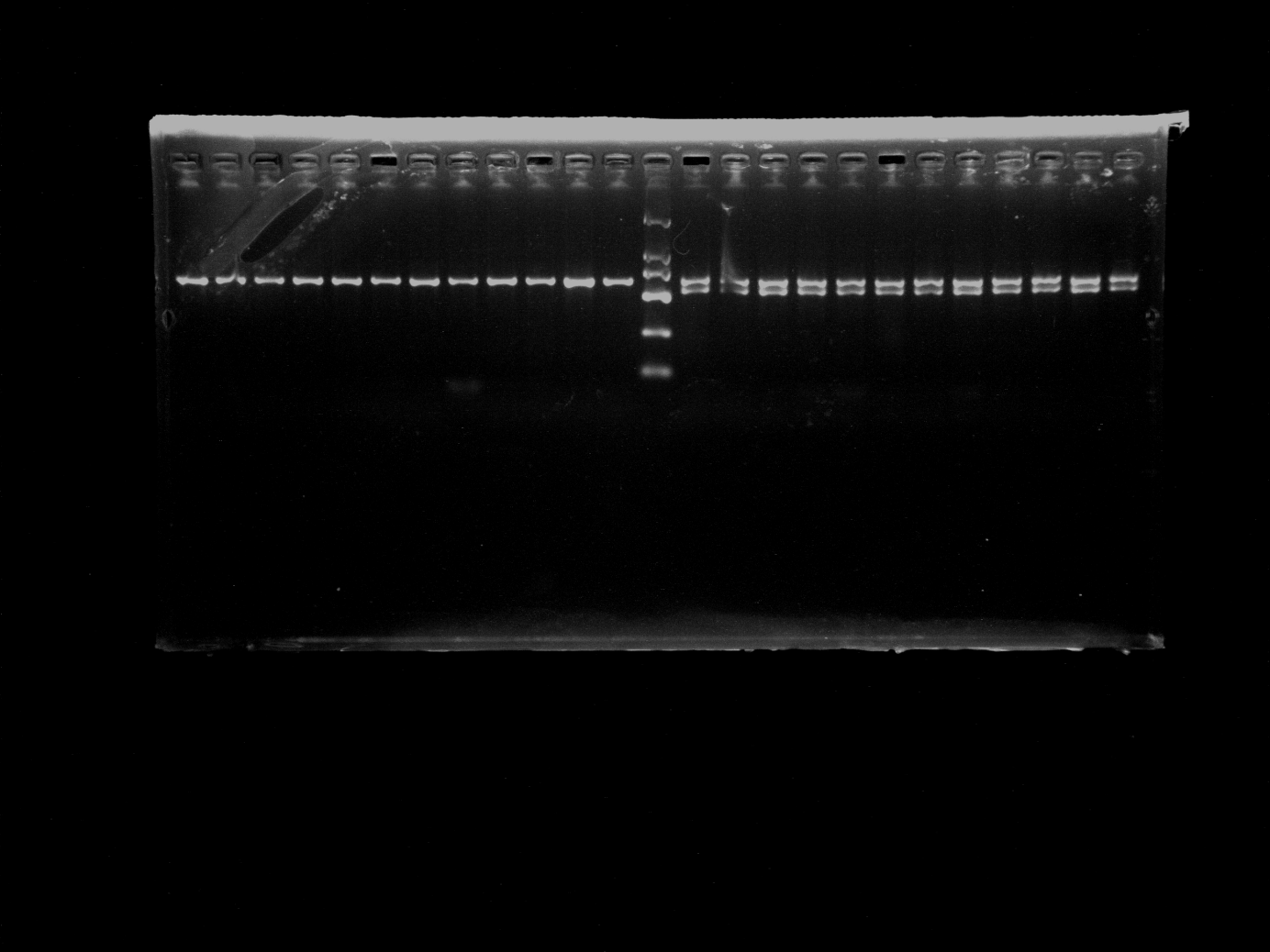


**Fig. S5.** The original gel electrophoresis image of PCR amplification results of 24 individuals from Taihu Lake. The DL 2000 DNA marker is shown in the middle. The 12 male individuals with a single band (641 bp) are shown on the left and the 12 female individuals with two bands (641 bp and 544 bp) are shown on the right.

## Supplementary Tables

## Table S1. Sample collection information and sequencing volume of all individuals resequenced in this study.

| **Id** | **Sex** | **Location** | **Longitude** | **Latitude** | **Sample date** | **Clean Data (Gb)** | **Depth** |
| --- | --- | --- | --- | --- | --- | --- | --- |
| HZ201 | male | Hongze Lake | 118.82°E | 33.28°N | 2022.01 | 12.17 | 27.0 |
| HZ202 | male | Hongze Lake | 118.82°E | 33.28°N | 2022.01 | 16.83 | 37.4 |
| HZ204 | male | Hongze Lake | 118.82°E | 33.28°N | 2022.01 | 16.05 | 35.7 |
| HZ206 | male | Hongze Lake | 118.82°E | 33.28°N | 2022.01 | 16.24 | 36.1 |
| HZ208 | male | Hongze Lake | 118.82°E | 33.28°N | 2022.01 | 15.56 | 34.6 |
| HZ209 | male | Hongze Lake | 118.82°E | 33.28°N | 2022.01 | 14.03 | 31.2 |
| HZ210 | male | Hongze Lake | 118.82°E | 33.28°N | 2022.01 | 14.45 | 32.1 |
| HZ211 | male | Hongze Lake | 118.82°E | 33.28°N | 2022.01 | 14.85 | 33.0 |
| TH201 | male | Taihu Lake | 120.32°E | 31.27°N | 2022.12 | 9.73 | 21.6 |
| TH202 | male | Taihu Lake | 120.32°E | 31.27°N | 2022.12 | 10.45 | 23.2 |
| TH203 | male | Taihu Lake | 120.32°E | 31.27°N | 2022.12 | 9.00 | 20.0 |
| TH204 | male | Taihu Lake | 120.32°E | 31.27°N | 2022.12 | 9.94 | 22.1 |
| TH205 | male | Taihu Lake | 120.32°E | 31.27°N | 2022.12 | 10.00 | 22.2 |
| TH206 | male | Taihu Lake | 120.32°E | 31.27°N | 2022.12 | 10.60 | 23.6 |
| TH207 | male | Taihu Lake | 120.32°E | 31.27°N | 2022.12 | 10.21 | 22.7 |
| TH208 | male | Taihu Lake | 120.32°E | 31.27°N | 2022.12 | 11.32 | 25.2 |
| TH209 | male | Taihu Lake | 120.32°E | 31.27°N | 2022.12 | 11.16 | 24.8 |
| TH210 | male | Taihu Lake | 120.32°E | 31.27°N | 2022.12 | 11.37 | 25.3 |
| TH211 | male | Taihu Lake | 120.32°E | 31.27°N | 2022.12 | 11.18 | 24.8 |
| TH212 | male | Taihu Lake | 120.32°E | 31.27°N | 2022.12 | 10.14 | 22.5 |
| HZ203 | female | Hongze Lake | 118.82°E | 33.28°N | 2022.01 | 15.80 | 35.1 |
| HZ205 | female | Hongze Lake | 118.82°E | 33.28°N | 2022.01 | 18.79 | 41.8 |
| HZ207 | female | Hongze Lake | 118.82°E | 33.28°N | 2022.01 | 16.73 | 37.2 |
| HZ214 | female | Hongze Lake | 118.82°E | 33.28°N | 2022.01 | 16.30 | 36.2 |
| HZ215 | female | Hongze Lake | 118.82°E | 33.28°N | 2022.01 | 17.93 | 39.8 |
| HZ216 | female | Hongze Lake | 118.82°E | 33.28°N | 2022.01 | 15.09 | 33.5 |
| HZ217 | female | Hongze Lake | 118.82°E | 33.28°N | 2022.01 | 17.10 | 38.0 |
| HZ218 | female | Hongze Lake | 118.82°E | 33.28°N | 2022.01 | 16.47 | 36.6 |
| HZ219 | female | Hongze Lake | 118.82°E | 33.28°N | 2022.01 | 16.10 | 35.8 |
| HZ220 | female | Hongze Lake | 118.82°E | 33.28°N | 2022.01 | 14.78 | 32.8 |
| HZ221 | female | Hongze Lake | 118.82°E | 33.28°N | 2022.01 | 17.98 | 40.0 |
| HZ222 | female | Hongze Lake | 118.82°E | 33.28°N | 2022.01 | 15.80 | 35.1 |
| TH213 | female | Taihu Lake | 120.32°E | 31.27°N | 2022.12 | 11.14 | 24.8 |
| TH214 | female | Taihu Lake | 120.32°E | 31.27°N | 2022.12 | 10.28 | 22.8 |
| TH215 | female | Taihu Lake | 120.32°E | 31.27°N | 2022.12 | 10.33 | 23.0 |
| TH216 | female | Taihu Lake | 120.32°E | 31.27°N | 2022.12 | 9.65 | 21.4 |
| TH217 | female | Taihu Lake | 120.32°E | 31.27°N | 2022.12 | 10.54 | 23.4 |
| TH218 | female | Taihu Lake | 120.32°E | 31.27°N | 2022.12 | 11.35 | 25.2 |
| TH219 | female | Taihu Lake | 120.32°E | 31.27°N | 2022.12 | 10.66 | 23.7 |
| TH220 | female | Taihu Lake | 120.32°E | 31.27°N | 2022.12 | 10.13 | 22.5 |
| TH221 | female | Taihu Lake | 120.32°E | 31.27°N | 2022.12 | 11.38 | 25.3 |
| TH222 | female | Taihu Lake | 120.32°E | 31.27°N | 2022.12 | 10.86 | 24.1 |
| TH223 | female | Taihu Lake | 120.32°E | 31.27°N | 2022.12 | 10.17 | 22.6 |
| TH224 | female | Taihu Lake | 120.32°E | 31.27°N | 2022.12 | 11.45 | 25.4 |

## Table S2. Summary of statistics for 46 sex-specific SNPs.

| **Chromosome** | **Position (bp)** | **p_value** | **F_ST_** | **Female_H_O_** | **Male_H_O_** |
| --- | --- | --- | --- | --- | --- |
| chr02 | 2,383,593 | 4.19E-08 | 0.4543 | 0.8750 | 0.0000 |
| chr02 | 2,396,438 | 1.55E-08 | 0.4783 | 1.0000 | 0.0000 |
| chr02 | 2,396,532 | 1.55E-08 | 0.4783 | 1.0000 | 0.0000 |
| chr02 | 2,396,565 | 1.55E-08 | 0.4783 | 1.0000 | 0.0000 |
| chr02 | 2,396,587 | 1.55E-08 | 0.4783 | 1.0000 | 0.0000 |
| chr02 | 2,396,642 | 1.55E-08 | 0.4783 | 1.0000 | 0.0000 |
| chr02 | 2,396,643 | 1.55E-08 | 0.4783 | 1.0000 | 0.0000 |
| chr02 | 2,396,666 | 1.55E-08 | 0.4783 | 1.0000 | 0.0000 |
| chr02 | 2396796 | 2.92E-07 | 0.4131 | 0.8750 | 0.0000 |
| chr02 | 2,396,825 | 1.55E-08 | 0.3916 | 0.8333 | 0.0000 |
| chr02 | 2,396,977 | 1.55E-08 | 0.4783 | 1.0000 | 0.0000 |
| chr02 | 2,396,985 | 1.55E-08 | 0.4783 | 1.0000 | 0.0000 |
| chr02 | 2,396,995 | 1.55E-08 | 0.4783 | 1.0000 | 0.0000 |
| chr02 | 2,397,264 | 1.55E-08 | 0.4783 | 1.0000 | 0.0000 |
| chr02 | 2,397,302 | 1.55E-08 | 0.4783 | 1.0000 | 0.0000 |
| chr02 | 2,397,561 | 1.55E-08 | 0.4783 | 1.0000 | 0.0000 |
| chr02 | 2,397,607 | 1.55E-08 | 0.4783 | 1.0000 | 0.0000 |
| chr02 | 2,397,626 | 1.55E-08 | 0.4783 | 1.0000 | 0.0000 |
| chr02 | 2,397,631 | 1.55E-08 | 0.4783 | 1.0000 | 0.0000 |
| chr02 | 2,397,803 | 3.42E-07 | 0.4588 | 1.0000 | 0.0000 |
| chr02 | 2,397,810 | 2.86E-08 | 0.4651 | 1.0000 | 0.0000 |
| chr02 | 2,397,858 | 1.55E-08 | 0.4831 | 1.0000 | 0.0000 |
| chr02 | 2,397,869 | 2.27E-08 | 0.5040 | 0.9546 | 0.0000 |
| chr02 | 2,397,887 | 3.66E-08 | 0.4919 | 0.9565 | 0.0000 |
| chr02 | 2,397,907 | 5.39E-08 | 0.4804 | 0.9583 | 0.0000 |
| chr02 | 2,420,160 | 4.68E-08 | 0.4725 | 1.0000 | 0.0000 |
| chr02 | 2,420,246 | 1.55E-08 | 0.4783 | 1.0000 | 0.0000 |
| chr02 | 2,420,393 | 1.55E-08 | 0.4783 | 1.0000 | 0.0000 |
| chr02 | 2,420,468 | 1.55E-08 | 0.4783 | 1.0000 | 0.0000 |
| chr02 | 2,420,509 | 1.55E-08 | 0.4783 | 1.0000 | 0.0000 |
| chr02 | 2,420,574 | 1.55E-08 | 0.4783 | 1.0000 | 0.0000 |
| chr02 | 2,420,702 | 1.55E-08 | 0.4783 | 1.0000 | 0.0000 |
| chr02 | 2,420,733 | 1.55E-08 | 0.4783 | 1.0000 | 0.0000 |
| chr02 | 2,420,735 | 1.55E-08 | 0.4783 | 1.0000 | 0.0000 |
| chr02 | 2,420,795 | 1.55E-08 | 0.4783 | 1.0000 | 0.0000 |
| chr02 | 2,420,838 | 1.55E-08 | 0.4783 | 1.0000 | 0.0000 |
| chr02 | 2,420,842 | 1.55E-08 | 0.4783 | 1.0000 | 0.0000 |
| chr02 | 2,421,063 | 1.55E-08 | 0.4783 | 1.0000 | 0.0000 |
| chr02 | 2,421,185 | 1.55E-08 | 0.4783 | 1.0000 | 0.0000 |
| chr02 | 2,421,194 | 4.19E-08 | 0.4565 | 0.9583 | 0.0000 |
| chr02 | 2,421,217 | 1.55E-08 | 0.4783 | 1.0000 | 0.0000 |
| chr02 | 2,421,242 | 1.55E-08 | 0.4783 | 1.0000 | 0.0000 |
| chr02 | 2,421,287 | 1.55E-08 | 0.4783 | 1.0000 | 0.0000 |
| chr02 | 2,421,658 | 1.55E-08 | 0.4783 | 1.0000 | 0.0000 |
| chr02 | 2,421,660 | 1.55E-08 | 0.4783 | 1.0000 | 0.0000 |
| chr02 | 2,421,767 | 3.01E-10 | 0.5764 | 0.8182 | 0.0000 |

The *p*_value is calculated by Fisher's exact test; Female_*H*_O_: observed heterozygosity in females; Male_*H*_O_: observed heterozygosity in males.

## Table S3. Depth of coverage for the 46 sex-specific SNPs in males and females.

| **Chromosome** | **Position (bp)** | **Welch’s t-test *p*** | **Male_average depth** | **Male_sd** | **Female_average depth** | **Female_sd** |
| --- | --- | --- | --- | --- | --- | --- |
| chr02 | 2,383,593 | 0.6033 | 27.05 | 6.5008 | 28.25 | 8.6791 |
| chr02 | 2,396,438 | 0.0681 | 30.70 | 6.5943 | 35.04 | 8.7600 |
| chr02 | 2,396,532 | 0.0125 | 30.20 | 10.0032 | 39.67 | 13.9710 |
| chr02 | 2,396,565 | 0.0645 | 35.85 | 11.7799 | 43.75 | 15.7735 |
| chr02 | 2,396,587 | 0.1165 | 35.70 | 11.9961 | 42.13 | 14.5969 |
| chr02 | 2,396,642 | 0.2289 | 37.70 | 14.0754 | 43.21 | 15.8306 |
| chr02 | 2,396,643 | 0.1716 | 37.55 | 13.3080 | 43.71 | 16.0610 |
| chr02 | 2,396,666 | 0.1769 | 36.70 | 13.9363 | 42.79 | 15.4610 |
| chr02 | 2396796 | 0.0503 | 37.50 | 13.6363 | 45.54 | 12.5420 |
| chr02 | 2,396,825 | 0.0314 | 35.40 | 13.4845 | 44.13 | 12.1666 |
| chr02 | 2,396,977 | 0.0998 | 38.50 | 16.5323 | 46.33 | 13.7419 |
| chr02 | 2,396,985 | 0.0601 | 36.20 | 14.5226 | 44.38 | 13.2166 |
| chr02 | 2,396,995 | 0.1421 | 39.85 | 16.5475 | 46.79 | 13.6094 |
| chr02 | 2,397,264 | 0.9480 | 39.95 | 14.8021 | 39.67 | 13.5668 |
| chr02 | 2,397,302 | 0.0631 | 24.65 | 8.9928 | 29.58 | 7.8956 |
| chr02 | 2,397,561 | 0.9834 | 41.90 | 17.4624 | 42.00 | 13.4714 |
| chr02 | 2,397,607 | 0.6365 | 40.80 | 16.2338 | 43.13 | 15.9954 |
| chr02 | 2,397,626 | 0.4336 | 41.90 | 17.8559 | 46.17 | 17.7707 |
| chr02 | 2,397,631 | 0.6161 | 42.50 | 18.6223 | 45.29 | 17.7898 |
| chr02 | 2,397,803 | 0.2868 | 36.20 | 13.3677 | 40.96 | 15.8868 |
| chr02 | 2,397,810 | 0.3243 | 36.10 | 12.2384 | 40.00 | 13.6827 |
| chr02 | 2,397,858 | 0.4258 | 39.20 | 15.3780 | 43.13 | 16.9662 |
| chr02 | 2,397,869 | 0.1671 | 35.05 | 14.6268 | 41.67 | 16.5731 |
| chr02 | 2,397,887 | 0.1242 | 31.60 | 14.7055 | 38.83 | 15.8297 |
| chr02 | 2,397,907 | 0.0465 | 28.50 | 13.3909 | 37.54 | 15.8498 |
| chr02 | 2,420,160 | 0.0264 | 21.95 | 5.4239 | 27.08 | 9.1267 |
| chr02 | 2,420,246 | 0.0044 | 27.50 | 7.7017 | 35.79 | 10.5252 |
| chr02 | 2,420,393 | 0.1380 | 32.80 | 12.6516 | 39.00 | 14.5363 |
| chr02 | 2,420,468 | 0.3692 | 39.90 | 17.2227 | 44.83 | 18.7864 |
| chr02 | 2,420,509 | 0.5223 | 39.75 | 17.3566 | 43.25 | 18.5595 |
| chr02 | 2,420,574 | 0.5255 | 39.00 | 14.5240 | 42.08 | 17.4229 |
| chr02 | 2,420,702 | 0.4796 | 38.00 | 14.0075 | 41.08 | 14.5868 |
| chr02 | 2,420,733 | 0.0749 | 32.50 | 11.3300 | 39.25 | 13.1785 |
| chr02 | 2,420,735 | 0.1066 | 32.90 | 11.5799 | 39.04 | 13.1132 |
| chr02 | 2,420,795 | 0.0513 | 31.70 | 9.1829 | 37.17 | 8.7460 |
| chr02 | 2,420,838 | 0.0164 | 31.95 | 8.4135 | 38.88 | 9.9665 |
| chr02 | 2,420,842 | 0.0188 | 31.70 | 8.5907 | 38.58 | 10.0862 |
| chr02 | 2,421,063 | 0.1648 | 29.25 | 9.7434 | 33.67 | 10.9650 |
| chr02 | 2,421,185 | 0.0575 | 33.90 | 14.6320 | 43.58 | 18.2469 |
| chr02 | 2,421,194 | 0.0706 | 35.05 | 15.9191 | 44.71 | 18.6045 |
| chr02 | 2,421,217 | 0.0333 | 33.40 | 17.3975 | 45.96 | 20.4503 |
| chr02 | 2,421,242 | 0.0612 | 36.05 | 17.2976 | 46.96 | 20.3224 |
| chr02 | 2,421,287 | 0.1487 | 35.60 | 14.8657 | 42.83 | 17.7486 |
| chr02 | 2,421,658 | 0.0038 | 30.00 | 8.9325 | 39.21 | 11.0019 |
| chr02 | 2,421,660 | 0.0086 | 30.30 | 9.1370 | 38.75 | 11.1910 |
| chr02 | 2,421,767 | 0.1071 | 33.75 | 11.7960 | 40.50 | 15.3708 |

## Table S4. Gene annotation results of 46 sex-specific SNPs.

| **Chromosome** | **Position (bp)** | **Gene ID** | **Gene name** | **Description** | **SNP effect** |
| --- | --- | --- | --- | --- | --- |
| chr02 | 2,383,593 | Nbr_001515-T1 | *MYO5C* | Unconventional Myosin-Vc | downstream_gene_variant |
| chr02 | 2,383,593 | Nbr_001516-T1 | *GNB5_1* | Guanine Nucleotide-binding Protein Subunit Beta-5 | intron_variant |
| chr02 | 2,383,593 | Nbr_001517-T1 | *AP4E1* | Adaptor Related Protein Complex 4 Subunit Epsilon 1 | upstream_gene_variant |
| chr02 | 2,396,438 | Nbr_001517-T1 | *AP4E1* | Adaptor Related Protein Complex 4 Subunit Epsilon 1 | downstream_gene_variant |
| chr02 | 2,396,532 | Nbr_001517-T1 | *AP4E1* | Adaptor Related Protein Complex 4 Subunit Epsilon 1 | downstream_gene_variant |
| chr02 | 2,396,565 | Nbr_001517-T1 | *AP4E1* | Adaptor Related Protein Complex 4 Subunit Epsilon 1 | downstream_gene_variant |
| chr02 | 2,396,587 | Nbr_001517-T1 | *AP4E1* | Adaptor Related Protein Complex 4 Subunit Epsilon 1 | downstream_gene_variant |
| chr02 | 2,396,642 | Nbr_001517-T1 | *AP4E1* | Adaptor Related Protein Complex 4 Subunit Epsilon 1 | downstream_gene_variant |
| chr02 | 2,396,643 | Nbr_001517-T1 | *AP4E1* | Adaptor Related Protein Complex 4 Subunit Epsilon 1 | downstream_gene_variant |
| chr02 | 2,396,666 | Nbr_001517-T1 | *AP4E1* | Adaptor Related Protein Complex 4 Subunit Epsilon 1 | downstream_gene_variant |
| chr02 | 2,396,796 | Nbr_001517-T1 | *AP4E1* | Adaptor Related Protein Complex 4 Subunit Epsilon 1 | downstream_gene_variant |
| chr02 | 2,396,825 | Nbr_001517-T1 | *AP4E1* | Adaptor Related Protein Complex 4 Subunit Epsilon 1 | downstream_gene_variant |
| chr02 | 2,396,977 | Nbr_001517-T1 | *AP4E1* | Adaptor Related Protein Complex 4 Subunit Epsilon 1 | downstream_gene_variant |
| chr02 | 2,396,985 | Nbr_001517-T1 | *AP4E1* | Adaptor Related Protein Complex 4 Subunit Epsilon 1 | downstream_gene_variant |
| chr02 | 2,396,995 | Nbr_001517-T1 | *AP4E1* | Adaptor Related Protein Complex 4 Subunit Epsilon 1 | downstream_gene_variant |
| chr02 | 2,397,264 | Nbr_001517-T1 | *AP4E1* | Adaptor Related Protein Complex 4 Subunit Epsilon 1 | downstream_gene_variant |
| chr02 | 2,397,302 | Nbr_001517-T1 | *AP4E1* | Adaptor Related Protein Complex 4 Subunit Epsilon 1 | downstream_gene_variant |
| chr02 | 2,396,438 | Nbr_001518-T1 | *CYP19B* | Cytochrome P450 Family 19 Subfamily B | synonymous_variant |
| chr02 | 2,396,532 | Nbr_001518-T1 | *CYP19B* | Cytochrome P450 Family 19 Subfamily B | upstream_gene_variant |
| chr02 | 2,396,565 | Nbr_001518-T1 | *CYP19B* | Cytochrome P450 Family 19 Subfamily B | upstream_gene_variant |
| chr02 | 2,396,587 | Nbr_001518-T1 | *CYP19B* | Cytochrome P450 Family 19 Subfamily B | upstream_gene_variant |
| chr02 | 2,396,642 | Nbr_001518-T1 | *CYP19B* | Cytochrome P450 Family 19 Subfamily B | upstream_gene_variant |
| chr02 | 2,396,643 | Nbr_001518-T1 | *CYP19B* | Cytochrome P450 Family 19 Subfamily B | upstream_gene_variant |
| chr02 | 2,396,666 | Nbr_001518-T1 | *CYP19B* | Cytochrome P450 Family 19 Subfamily B | upstream_gene_variant |
| chr02 | 2,396,796 | Nbr_001518-T1 | *CYP19B* | Cytochrome P450 Family 19 Subfamily B | upstream_gene_variant |
| chr02 | 2,396,825 | Nbr_001518-T1 | *CYP19B* | Cytochrome P450 Family 19 Subfamily B | upstream_gene_variant |
| chr02 | 2,396,977 | Nbr_001518-T1 | *CYP19B* | Cytochrome P450 Family 19 Subfamily B | upstream_gene_variant |
| chr02 | 2,396,985 | Nbr_001518-T1 | *CYP19B* | Cytochrome P450 Family 19 Subfamily B | upstream_gene_variant |
| chr02 | 2,396,995 | Nbr_001518-T1 | *CYP19B* | Cytochrome P450 Family 19 Subfamily B | upstream_gene_variant |
| chr02 | 2,397,264 | Nbr_001518-T1 | *CYP19B* | Cytochrome P450 Family 19 Subfamily B | upstream_gene_variant |
| chr02 | 2,397,302 | Nbr_001518-T1 | *CYP19B* | Cytochrome P450 Family 19 Subfamily B | upstream_gene_variant |
| chr02 | 2,397,561 | Nbr_001518-T1 | *CYP19B* | Cytochrome P450 Family 19 Subfamily B | upstream_gene_variant |
| chr02 | 2,397,607 | Nbr_001518-T1 | *CYP19B* | Cytochrome P450 Family 19 Subfamily B | upstream_gene_variant |
| chr02 | 2,397,626 | Nbr_001518-T1 | *CYP19B* | Cytochrome P450 Family 19 Subfamily B | upstream_gene_variant |
| chr02 | 2,397,631 | Nbr_001518-T1 | *CYP19B* | Cytochrome P450 Family 19 Subfamily B | upstream_gene_variant |
| chr02 | 2,397,803 | Nbr_001518-T1 | *CYP19B* | Cytochrome P450 Family 19 Subfamily B | upstream_gene_variant |
| chr02 | 2,397,810 | Nbr_001518-T1 | *CYP19B* | Cytochrome P450 Family 19 Subfamily B | upstream_gene_variant |
| chr02 | 2,397,858 | Nbr_001518-T1 | *CYP19B* | Cytochrome P450 Family 19 Subfamily B | upstream_gene_variant |
| chr02 | 2,397,869 | Nbr_001518-T1 | *CYP19B* | Cytochrome P450 Family 19 Subfamily B | upstream_gene_variant |
| chr02 | 2,397,887 | Nbr_001518-T1 | *CYP19B* | Cytochrome P450 Family 19 Subfamily B | upstream_gene_variant |
| chr02 | 2,397,907 | Nbr_001518-T1 | *CYP19B* | Cytochrome P450 Family 19 Subfamily B | upstream_gene_variant |
| chr02 | 2,396,438 | Nbr_001519-T1 | *PCLOL1* | Piccolo Presynaptic Cytomatrix Protein Like 1 | upstream_gene_variant |
| chr02 | 2,396,532 | Nbr_001519-T1 | *PCLOL1* | Piccolo Presynaptic Cytomatrix Protein Like 1 | upstream_gene_variant |
| chr02 | 2,396,565 | Nbr_001519-T1 | *PCLOL1* | Piccolo Presynaptic Cytomatrix Protein Like 1 | upstream_gene_variant |
| chr02 | 2,396,587 | Nbr_001519-T1 | *PCLOL1* | Piccolo Presynaptic Cytomatrix Protein Like 1 | upstream_gene_variant |
| chr02 | 2,396,642 | Nbr_001519-T1 | *PCLOL1* | Piccolo Presynaptic Cytomatrix Protein Like 1 | upstream_gene_variant |
| chr02 | 2,396,643 | Nbr_001519-T1 | *PCLOL1* | Piccolo Presynaptic Cytomatrix Protein Like 1 | upstream_gene_variant |
| chr02 | 2,396,666 | Nbr_001519-T1 | *PCLOL1* | Piccolo Presynaptic Cytomatrix Protein Like 1 | upstream_gene_variant |
| chr02 | 2,396,796 | Nbr_001519-T1 | *PCLOL1* | Piccolo Presynaptic Cytomatrix Protein Like 1 | upstream_gene_variant |
| chr02 | 2,396,825 | Nbr_001519-T1 | *PCLOL1* | Piccolo Presynaptic Cytomatrix Protein Like 1 | upstream_gene_variant |
| chr02 | 2,396,977 | Nbr_001519-T1 | *PCLOL1* | Piccolo Presynaptic Cytomatrix Protein Like 1 | upstream_gene_variant |
| chr02 | 2,396,985 | Nbr_001519-T1 | *PCLOL1* | Piccolo Presynaptic Cytomatrix Protein Like 1 | upstream_gene_variant |
| chr02 | 2,396,995 | Nbr_001519-T1 | *PCLOL1* | Piccolo Presynaptic Cytomatrix Protein Like 1 | upstream_gene_variant |
| chr02 | 2,397,264 | Nbr_001519-T1 | *PCLOL1* | Piccolo Presynaptic Cytomatrix Protein Like 1 | upstream_gene_variant |
| chr02 | 2,397,302 | Nbr_001519-T1 | *PCLOL1* | Piccolo Presynaptic Cytomatrix Protein Like 1 | upstream_gene_variant |
| chr02 | 2,397,561 | Nbr_001519-T1 | *PCLOL1* | Piccolo Presynaptic Cytomatrix Protein Like 1 | upstream_gene_variant |
| chr02 | 2,397,607 | Nbr_001519-T1 | *PCLOL1* | Piccolo Presynaptic Cytomatrix Protein Like 1 | upstream_gene_variant |
| chr02 | 2,397,626 | Nbr_001519-T1 | *PCLOL1* | Piccolo Presynaptic Cytomatrix Protein Like 1 | upstream_gene_variant |
| chr02 | 2,397,631 | Nbr_001519-T1 | *PCLOL1* | Piccolo Presynaptic Cytomatrix Protein Like 1 | upstream_gene_variant |
| chr02 | 2,397,803 | Nbr_001519-T1 | *PCLOL1* | Piccolo Presynaptic Cytomatrix Protein Like 1 | upstream_gene_variant |
| chr02 | 2,397,810 | Nbr_001519-T1 | *PCLOL1* | Piccolo Presynaptic Cytomatrix Protein Like 1 | upstream_gene_variant |
| chr02 | 2,397,858 | Nbr_001519-T1 | *PCLOL1* | Piccolo Presynaptic Cytomatrix Protein Like 1 | upstream_gene_variant |
| chr02 | 2,397,869 | Nbr_001519-T1 | *PCLOL1* | Piccolo Presynaptic Cytomatrix Protein Like 1 | upstream_gene_variant |
| chr02 | 2,397,887 | Nbr_001519-T1 | *PCLOL1* | Piccolo Presynaptic Cytomatrix Protein Like 1 | upstream_gene_variant |
| chr02 | 2,397,907 | Nbr_001519-T1 | *PCLOL1* | Piccolo Presynaptic Cytomatrix Protein Like 1 | upstream_gene_variant |
| chr02 | 2,396,438 | Nbr_001520-T1 | *PCLOL2* | Piccolo Presynaptic Cytomatrix Protein Like 2 | upstream_gene_variant |
| chr02 | 2,396,532 | Nbr_001520-T1 | *PCLOL2* | Piccolo Presynaptic Cytomatrix Protein Like 2 | upstream_gene_variant |
| chr02 | 2,396,565 | Nbr_001520-T1 | *PCLOL2* | Piccolo Presynaptic Cytomatrix Protein Like 2 | upstream_gene_variant |
| chr02 | 2,396,587 | Nbr_001520-T1 | *PCLOL2* | Piccolo Presynaptic Cytomatrix Protein Like 2 | upstream_gene_variant |
| chr02 | 2,396,642 | Nbr_001520-T1 | *PCLOL2* | Piccolo Presynaptic Cytomatrix Protein Like 2 | upstream_gene_variant |
| chr02 | 2,396,643 | Nbr_001520-T1 | *PCLOL2* | Piccolo Presynaptic Cytomatrix Protein Like 2 | upstream_gene_variant |
| chr02 | 2,396,666 | Nbr_001520-T1 | *PCLOL2* | Piccolo Presynaptic Cytomatrix Protein Like 2 | upstream_gene_variant |
| chr02 | 2,396,796 | Nbr_001520-T1 | *PCLOL2* | Piccolo Presynaptic Cytomatrix Protein Like 2 | upstream_gene_variant |
| chr02 | 2,396,825 | Nbr_001520-T1 | *PCLOL2* | Piccolo Presynaptic Cytomatrix Protein Like 2 | upstream_gene_variant |
| chr02 | 2,396,977 | Nbr_001520-T1 | *PCLOL2* | Piccolo Presynaptic Cytomatrix Protein Like 2 | upstream_gene_variant |
| chr02 | 2,396,985 | Nbr_001520-T1 | *PCLOL2* | Piccolo Presynaptic Cytomatrix Protein Like 2 | upstream_gene_variant |
| chr02 | 2,396,995 | Nbr_001520-T1 | *PCLOL2* | Piccolo Presynaptic Cytomatrix Protein Like 2 | upstream_gene_variant |
| chr02 | 2,397,264 | Nbr_001520-T1 | *PCLOL2* | Piccolo Presynaptic Cytomatrix Protein Like 2 | upstream_gene_variant |
| chr02 | 2,397,302 | Nbr_001520-T1 | *PCLOL2* | Piccolo Presynaptic Cytomatrix Protein Like 2 | upstream_gene_variant |
| chr02 | 2,397,561 | Nbr_001520-T1 | *PCLOL2* | Piccolo Presynaptic Cytomatrix Protein Like 2 | upstream_gene_variant |
| chr02 | 2,397,607 | Nbr_001520-T1 | *PCLOL2* | Piccolo Presynaptic Cytomatrix Protein Like 2 | upstream_gene_variant |
| chr02 | 2,397,626 | Nbr_001520-T1 | *PCLOL2* | Piccolo Presynaptic Cytomatrix Protein Like 2 | upstream_gene_variant |
| chr02 | 2,397,631 | Nbr_001520-T1 | *PCLOL2* | Piccolo Presynaptic Cytomatrix Protein Like 2 | upstream_gene_variant |
| chr02 | 2,397,803 | Nbr_001520-T1 | *PCLOL2* | Piccolo Presynaptic Cytomatrix Protein Like 2 | upstream_gene_variant |
| chr02 | 2,397,810 | Nbr_001520-T1 | *PCLOL2* | Piccolo Presynaptic Cytomatrix Protein Like 2 | upstream_gene_variant |
| chr02 | 2,397,858 | Nbr_001520-T1 | *PCLOL2* | Piccolo Presynaptic Cytomatrix Protein Like 2 | upstream_gene_variant |
| chr02 | 2,397,869 | Nbr_001520-T1 | *PCLOL2* | Piccolo Presynaptic Cytomatrix Protein Like 2 | upstream_gene_variant |
| chr02 | 2,397,887 | Nbr_001520-T1 | *PCLOL2* | Piccolo Presynaptic Cytomatrix Protein Like 2 | upstream_gene_variant |
| chr02 | 2,397,907 | Nbr_001520-T1 | *PCLOL2* | Piccolo Presynaptic Cytomatrix Protein Like 2 | upstream_gene_variant |
| chr02 | 2,420,160 | Nbr_001521-T1 | *CLPX* | Caseinolytic Mitochondrial Matrix Peptidase Chaperone Subunit X | 5_prime_UTR_variant |
| chr02 | 2,420,246 | Nbr_001521-T1 | *CLPX* | Caseinolytic Mitochondrial Matrix Peptidase Chaperone Subunit X | 5_prime_UTR_variant |
| chr02 | 2,420,393 | Nbr_001521-T1 | *CLPX* | Caseinolytic Mitochondrial Matrix Peptidase Chaperone Subunit X | 5_prime_UTR_variant |
| chr02 | 2,420,468 | Nbr_001521-T1 | *CLPX* | Caseinolytic Mitochondrial Matrix Peptidase Chaperone Subunit X | 5_prime_UTR_variant |
| chr02 | 2,420,509 | Nbr_001521-T1 | *CLPX* | Caseinolytic Mitochondrial Matrix Peptidase Chaperone Subunit X | 5_prime_UTR_variant |
| chr02 | 2,420,574 | Nbr_001521-T1 | *CLPX* | Caseinolytic Mitochondrial Matrix Peptidase Chaperone Subunit X | 5_prime_UTR_variant |
| chr02 | 2,420,702 | Nbr_001521-T1 | *CLPX* | Caseinolytic Mitochondrial Matrix Peptidase Chaperone Subunit X | intron_variant |
| chr02 | 2,420,733 | Nbr_001521-T1 | *CLPX* | Caseinolytic Mitochondrial Matrix Peptidase Chaperone Subunit X | intron_variant |
| chr02 | 2,420,735 | Nbr_001521-T1 | *CLPX* | Caseinolytic Mitochondrial Matrix Peptidase Chaperone Subunit X | intron_variant |
| chr02 | 2,420,795 | Nbr_001521-T1 | *CLPX* | Caseinolytic Mitochondrial Matrix Peptidase Chaperone Subunit X | intron_variant |
| chr02 | 2,420,838 | Nbr_001521-T1 | *CLPX* | Caseinolytic Mitochondrial Matrix Peptidase Chaperone Subunit X | intron_variant |
| chr02 | 2,420,842 | Nbr_001521-T1 | *CLPX* | Caseinolytic Mitochondrial Matrix Peptidase Chaperone Subunit X | intron_variant |
| chr02 | 2,421,063 | Nbr_001521-T1 | *CLPX* | Caseinolytic Mitochondrial Matrix Peptidase Chaperone Subunit X | intron_variant |
| chr02 | 2,421,185 | Nbr_001521-T1 | *CLPX* | Caseinolytic Mitochondrial Matrix Peptidase Chaperone Subunit X | intron_variant |
| chr02 | 2,421,194 | Nbr_001521-T1 | *CLPX* | Caseinolytic Mitochondrial Matrix Peptidase Chaperone Subunit X | intron_variant |
| chr02 | 2,421,217 | Nbr_001521-T1 | *CLPX* | Caseinolytic Mitochondrial Matrix Peptidase Chaperone Subunit X | intron_variant |
| chr02 | 2,421,242 | Nbr_001521-T1 | *CLPX* | Caseinolytic Mitochondrial Matrix Peptidase Chaperone Subunit X | intron_variant |
| chr02 | 2,421,287 | Nbr_001521-T1 | *CLPX* | Caseinolytic Mitochondrial Matrix Peptidase Chaperone Subunit X | intron_variant |
| chr02 | 2,421,658 | Nbr_001521-T1 | *CLPX* | Caseinolytic Mitochondrial Matrix Peptidase Chaperone Subunit X | intron_variant |
| chr02 | 2,421,660 | Nbr_001521-T1 | *CLPX* | Caseinolytic Mitochondrial Matrix Peptidase Chaperone Subunit X | intron_variant |
| chr02 | 2,421,767 | Nbr_001521-T1 | *CLPX* | Caseinolytic Mitochondrial Matrix Peptidase Chaperone Subunit X | intron_variant |

## Table S5. Distribution of the four small insertions on the Z or W chromosomes in the sex determining region.

| **ID** | **Chromosome** | **Position** | **Reference** | **Alternate** | **Z_ref** | **Z_alt** | **W_ref** | **W_alt** |
| --- | --- | --- | --- | --- | --- | --- | --- | --- |
| pbsv_5698 | chr02 | 2,392,668 | G | GTGCTGAAACATAAACTGTCATGTCTTAAAGTATTATATTTCATATGGA | 0/47 | 26/47 | 20/47 | 1/47 |
| pbsv_5699 | chr02 | 2,393,003 | A | AAGTAAGAAAGTAAATTTTTTT | 27/49 | 0/49 | 1/49 | 21/49 |
| pbsv_5700 | chr02 | 2,394,956 | C | CCCCCCCCCCCCACACACACACACACACACACACACACACACACACA | 0/64 | 32/64 | 31/64 | 1/64 |
| pbsv_5701 | chr02 | 2,396,484 | G | GGGCACAGATGTTGAAGTGATAAGGTCGGGTCAGGCGGATCAGG | 0/76 | 38/76 | 37/76 | 1/76 |

The denominators represent the number of reads where insertions and sex-specific SNP (chr02_2396438) occur simultaneously. The molecules represent the number of reference and alternate distributed on the W or Z chromosome.
